# Hepatitis C virus seroprevalence in the general female population of 9 countries in Europe, Asia and Africa

**DOI:** 10.1186/s13027-017-0121-1

**Published:** 2017-02-02

**Authors:** Gary M. Clifford, Tim Waterboer, Bolormaa Dondog, You Lin Qiao, Dimitri Kordzaia, Doudja Hammouda, Namory Keita, Nahid Khodakarami, Syed Ahsan Raza, Ang Tshering Sherpa, Witold Zatonski, Michael Pawlita, Martyn Plummer, Silvia Franceschi

**Affiliations:** 1 International Agency for Research on Cancer, 150 cours Albert Thomas, 69372 Lyon Cedex 08, France; 2 Infection, Inflammation and Cancer Program, German Cancer Research Center (DKFZ), Heidelberg, Germany; 3 Cancer Institute of the Chinese Academy of Medical Sciences, Beijing, China; 4 Iv. Javakhishvili Tbilisi State University, Tbilisi, Georgia; 5 Institut National de Sante Publique, Algiers, Algeria; 6 Department of Obstetrics and Gynaecology, Centre Hospitalier Universitaire de Donka, Conakry, Guinea; 7 Infertility and Reproductive Health Research Centre, Shahid Beheshti University of Medical Sciences, Tehran, Iran; 8 Department of Surgery, The Aga Khan University, Karachi, Pakistan; 9 Centre de Recherche du CHUM, Département de Médecine Sociale et Préventive Université de Montréal, Quebec, Canada; 10 Kist Medical College, Lalitpur, Nepal; 11 The Maria Sklodowska-Curie Memorial Cancer Center and Institute of Oncology, Warsaw, Poland

**Keywords:** Hepatitis C virus, Epidemiology, Serology, Liver cancer

## Abstract

**Background:**

New oral treatments with very high cure rates have the potential to revolutionize global management of hepatitis C virus (HCV), but population-based data on HCV infection are missing in many low and middle-income countries (LMIC).

**Methods:**

Between 2004 and 2009, dried blood spots were collected from age-stratified female population samples of 9 countries: China, Mongolia, Poland, Guinea, Nepal, Pakistan, Algeria, Georgia and Iran. HCV antibodies were detected by a multiplex serology assay using bead-based technology.

**Results:**

Crude HCV prevalence ranged from 17.4% in Mongolia to 0.0% in Iran. In a pooled model adjusted by age and country, in which associations with risk factors were not statistically heterogeneous across countries, the only significant determinants of HCV positivity were age (prevalence ratio for ≥45 versus <35 years = 2.84, 95%CI 2.18-3.71) and parity (parous versus nulliparous = 1.73, 95%CI 1.02-2.93). Statistically significant increases in HCV positivity by age, but not parity, were seen in each of the three countries with the highest number of HCV infections: Mongolia, Pakistan, China. There were no associations with sexual partners nor HPV infection. HCV prevalence in women aged ≥45 years correlated well with recent estimates of female HCV-related liver cancer incidence, with the slight exception of Pakistan, which showed a higher HCV prevalence (5.2%) than expected.

**Conclusions:**

HCV prevalence varies enormously in women worldwide. Medical interventions/hospitalizations linked to childbirth may have represented a route of HCV transmission, but not sexual intercourse. Combining dried blood spot collection with high-throughput HCV assays can facilitate seroepidemiological studies in LMIC where data is otherwise scarce.

## Background

About 180 million people, some 3% of the world’s population, are estimated to have been exposed to hepatitis C virus (HCV) [1–3]. Of these, 130–150 million (~80%) are chronically HCV infected and at risk for development of hepatitis-C related liver cirrhosis or cancer, which kill approximately 700,000 people each year [4]. Although new HCV treatments offering very high cure rates with short durations and few side effects have the potential to drastically reduce HCV-related mortality, access to diagnosis and expensive treatment remain low, so that the number of people living with HCV is actually reported to be increasing [1].

In 2016, the World Health Assembly adopted a strategy to eliminate viral hepatitis as a major public health threat by 2030, noting that national data on hepatitis virus infection are often lacking [5]. In addition, available HCV surveys often over-represent low-risk groups, particularly younger low-risk persons (e.g. pregnant women and blood donors), whereas HCV prevalence is known to increase steadily with age owing to the combination of accumulating risk of exposure and a high probability of infection becoming chronic [6].

Common routes of HCV infection are unsafe injections, inadequate sterilization of medical equipment, and the transfusion of unscreened blood and blood products that have been common in high-income countries till the 1980s and also more recently in low- and medium-income countries (LMIC). Because these practices are largely specific to a given country’s medical system, HCV prevalence and the timing of infection spread may differ between bordering countries [2], and national level data on HCV infection is required to inform public health decisions.

Hence, we exploited a series of standardized seroepidemiological surveys in order to describe HCV prevalence and risk factors among the general female population of a heterogeneous range of countries around the world. HCV seropositivity was determined using a high throughput assay that has been shown to provide an accurate and cost-effective tool for assessment of HCV antibodies in large epidemiologic studies [7], and we went on to compare country-specific HCV prevalence with recently generated country-specific estimates of HCV-related liver cancer incidence.

## Methods

### Population

Between 2004 and 2009, studies were undertaken in 11 areas in 9 countries with the primary aim to estimate the prevalence of genital human papillomavirus virus (HPV) infection, according to a similar protocol developed and co-ordinated by the International Agency for Research on Cancer (IARC). Population sampling methods have been previously described for the individual study centres: Shanxi, China [8]; Shenyang, China [9]; Shenzhen, China [10]; Ulaanbaatar, Mongolia [11]; Warsaw, Poland [12]; Conakry, Guinea [13]; Bharatpur, Nepal [14]; Karachi, Pakistan [15]; Zeralda, Algeria [16]; Tbilisi, Georgia [17]; and Tehran, Iran [18]. In each area, an attempt was made to obtain an age-stratified population-based sample that included at least 100 women in each 5-year age group, from 15-19 up to 65 years and older. The number of included women is sometimes higher than that in the original reports due to relaxing of selection criteria for adequacy of genital specimens. Data from Mongolia have been previously reported in validation studies of the present HCV serological assay [7, 19]. Trained interviewers administered a face-to-face questionnaire that included information on sociodemographic characteristics, sexual behaviour, reproductive and contraceptive history, and smoking habits. All participants signed informed consent forms according to the recommendations of the IARC Ethics Committee, and of the local ethical review committees in each of the participating countries, which also approved each of the original studies.

### Specimen collection

Each participant had a blood sample collected by venopuncture. In Mongolia, this sample was drawn from the cubital fossa into vacuum containers without anticoagulant [19], whereas in all other areas, samples were obtained by fingerstick. In both instances, full blood was then immediately applied to DBS (dried blood spot) filter paper card (Whatman 903 Protein Saver Blood Collection Cards; Schleicher & Schuell) to entirely fill five 14.5 mm diameter circles. DBS cards were dried at room temperature, placed in separate plastic paper zip lock envelopes containing a silica desiccant, and shipped at ambient temperature to the German Cancer Research Center (DKFZ) in Heidelberg, Germany, and stored at -20°C until serological analysis.

Study participants were also invited for a pelvic examination (although a subset, particularly self-reported virgins, declined) and collection of cervical exfoliated cells for HPV DNA testing, performed in the Department of Pathology, VU University Medical Center, Amsterdam, the Netherlands, according to a general primer GP5+/6 + –mediated PCR [20].

### HCV serology

Antibodies were eluted from DBS cards according to a previously reported protocol that has been shown to result in high agreement (>96% for HCV antigens) of seropositivity in paired DBS and serum results [19]. In brief, one punch of 6 mm diameter from each DBS was eluted in one well of a 96-well plate in 100 μL PBS at 4^o^C overnight, and 16 μl of eluate subsequently mixed with 80 μL DBS preincubation buffer.

A multiplex serology assay [19] including antigens to HCV (strain H77, subtype 1a) Core and NS3 proteins was performed as previously described [7]. In brief, full-length coding sequences of Core and NS3 were expressed as double fusion proteins with an N-terminal-GST and a C-terminal tag epitope derived from the large T-antigen of SV40 in E. coli. Fusion proteins were loaded and affinity-purified on glutathione-casein coupled spectrally distinct fluorescence-labeled polystyrene beads (SeroMap; Luminex). DBS eluates were incubated with pooled antigen loaded bead sets. Bound antibodies were quantified with biotinylated goat anti-human IgA, IgM, IgG (Dianova), and R-phycoerythrin–labeled streptavidin in a Luminex 100 analyzer as the median R-phycoerythrin fluorescence intensity (MFI) from at least 100 beads of the same bead set. Antigen-specific MFI values were calculated as previously described [21].

DBS-specific cut-off values for HCV Core (MFI ≥ 967) and NS3 (MFI ≥ 310) were defined as the mean MFI plus three SDs from 235 reference sera that tested negative by a commercial HCV antibody screening assay [19]. Women that were positive for both Core and NS3 proteins were defined as being HCV-positive. In an evaluation against a set of 432 reference sera, this cut-off has been proven to perform similarly, in a single step, to a commercial HCV antibody screening assay (MEIA) followed by RNA confirmation (98% sensitivity, 99% specificity) [7].

When a subset of 2,988 DBS samples from 8 study areas were re-tested on a second occasion to evaluate assay reproducibility, 2,982 (99.8%) were concordantly classified (2,940 HCV-negative, 42 HCV-positive).

### Statistical analyses

HPV prevalence was standardized by age using the world standard population (in 5-year age groups from 15-19 to 60-65 years) as a reference [22]. Prevalence ratios (PR) for HCV seropositivity and corresponding 95% confidence intervals (CI) were calculated using unconditional logistic regression adjusted for age (5-year groups) and study area. Heterogeneity of PRs between study areas was tested by calculating the difference between the log likelihood of the model that considered the interaction term between the areas and risk factor of interest and the log likelihood of the model that included the exposure only, and comparing it to the chi-squared distribution with degrees of freedom equal to the number of areas minus one.

Country-specific estimates of HCV-related liver cancer incidence in women were extracted from Plummer et al, Lancet Global Health, 2016 [23], derived by combining country-specific estimates of HCV attributable fraction in liver cancer with Globocan 2012 [24] estimates of female liver cancer incidence.

## Results

HCV serology results were obtained for a total of 12,204 women, with study-specific sample sizes varying from 1,516 for Shenzhen, China, down to 892 for Iran (Table 1). Overall median age was 34 years, varying from 30 to 38 years by study center (Table 1). A total of 274 women (2.2%) were HCV-positive. Crude HCV prevalence ranged from 17.4% in Mongolia down to 0.0% in Iran (Table 1). Age-standardization reduced HCV prevalence estimates (by increasing the relative weight of younger age groups, who were less often HCV-positive), but did not change relative differences in HCV positivity between centers.

**Table 1:** HCV prevalence by study center

				HCV prevalence			
				Crude		Age-standardized^a^	
Geographical area	Median age	N tested	N pos	%	95% CI	%	95% CI
Mongolia, Ulaanbaatar	35	1,075	187	17.4	15.2-19.2	10.9	3.3-18.6
Pakistan, Karachi	35	963	31	3.2	2.2-4.5	1.7	0.5-2.9
Georgia, Tbilisi	33	1,431	17	1.2	0.7-1.9	0.7	0.0-1.4
Guinea, Conakry	30	1,253	11	0.9	0.4-1.6	0.5	0.0-1.4
Poland, Warsaw	37	909	7	0.8	0.3-1.6	0.3	0.0-0.9
China, Shenyang	34	989	6	0.6	0.2-1.3	0.4	0.0-1.7
China, Shanxi	36	940	6	0.6	0.2-1.4	0.3	0.0-0.8
China, Shenzhen	30	1,516	6	0.4	0.1-0.9	0.3	0.0-0.6
Nepal, Bharatpur	33	1,061	2	0.2	0.0-0.7	0.2	0.0-0.8
Algeria, Zeralda	36	1,135	1	0.1	0.0-0.5	0.1	0.0-0.2
Iran, Tehran	38	892	0	0	0.0-0.4	0.0	0.0-0.4
Total	34	12,164	274	2.2	2.0-2.5	1.4	1.0-1.83

^a^Standardized to world population in CI5 [22]

Table 2 shows the relationship of selected characteristics of study women with HCV positivity. In a model adjusted by age and study area, as appropriate, the only significant determinants of HCV positivity were age group (PR for ≥45 versus <35 years = 2.84, 95% CI 2.18-3.71) and parity (PR for parous versus nulliparous =1.73, 95% CI 1.02-2.93). However, no risk trend was found by number of births. Associations of HCV positivity with risk factors were not statistically heterogeneous across study areas (p values for heterogeneity = 0.17, 0.88, 0.59 and 0.25 for age, sexual partners, parity and induced abortion, respectively).

**Table 2:** Prevalence ratios (PR) for HCV positivity and corresponding 95% confidence intervals (CI) according to selected women’s characteristics

Risk factor	N tested	HCV positive N (%)	Adjusted PR^a^	95% CI
Age	12,164			
<35	6,249	79 (1.3)	1	-
35-44	2,894	78 (2.7)	1.86	1.39-2.50
≥45	3,021	117 (3.9)	2.84	2.18-3.71
*χ*_1_^2^ *for trend*			*p < 0.001*	
Education level	12,156			
None	1,713	24 (1.4)	1	-
Primary	1,552	21 (1.3)	0.96	0.52-1.77
Secondary and higher	8,891	229 (2.6)	0.86	0.46-1.60
Lifetime number of sexual partners^b^	10,926			
0	1,086	1 (0.1)	0.21	0.03-1.55
1	7,513	146 (1.9)	1	-
2	1,305	60 (4.6)	1.12	0.84-1.49
3+	1,022	65 (6.4)	1.08	0.81-1.44
*χ*_1_^2^ *for trend*			*p = 0.367*	
Number of births (parity)	11,199			
Nulliparous	2,207	21 (0.9)	1	-
1	3,228	55 (1.7)	1.68^d^	0.98-2.90
2	2,644	75 (2.8)	1.81^d^	1.01-3.26
3+	3,120	119 (3.8)	1.84^d^	1.01-3.38
* χ*_1_^2^ *for trend*			*p = 0.171*	
Induced abortion^c^	9,166			
0	5,433	95 (1.7)	1	-
1	1,750	46 (2.6)	0.97	0.69-1.37
2+	1,983	112 (5.6)	1.24	0.93-1.66
* χ*_1_^2^ *for trend*			*p = 0.121*	
Smoking status	12,153			
Never	10,860	235 (2.2)	1	-
Ever	1,293	38 (2.9)	1.21	0.88-1.66
HPV DNA-positive	9,984			
No	8,264	183 (2.2)	1	-
Yes	1,720	65 (3.8)	1.04	0.80-1.36

^a^Adjusted for age (5-year groups) and geographical area, as appropriate

^b^Algeria excluded because of missing data

^c^Algeria and Guinea excluded because of missing data

^d^Combined PR for ≥ 1 versus 0 = 1.73 (1.02-2.93)

Findings by age group and parity are also shown separately for Mongolia, Pakistan and China (the three countries with the highest number of HCV-positive women) in Table 3. Statistically significant increases in HCV positivity by age were seen in each of the three countries, but the rise between women <35 to those 35-44 years of age was especially steep in Pakistan. Increases in HCV positivity in parous women were only observed in Mongolia and Pakistan and did not meet statistical significance (Table 3).

**Table 3:** HCV positivity by age and number of births, separately for Mongolia, Pakistan and China

	Mongolia			Pakistan			China		
Risk factor	N tested	HCV positive N (%)	Adjusted PR^a^ and 95% CI	N tested	HCV positive N (%)	Adjusted PR^a^ and 95% CI	N tested	HCV positive N (%)	Adjusted PR^a^ and 95% CI
Age	1,075			963			3,445		
<35	511	55 (10.8)	1	479	7 (1.5)	1	1,915	5 (0.3)	1
35-44	289	54 (18.7)	1.74 (1.23-2.45)	254	12 (4.7)	3.23 (1.29-8.11)	755	4 (0.5)	2.03 (0.55-7.54)
≥45	275	78 (28.4)	2.63 (1.93-3.60)	230	12 (5.2)	3.57 (1.42-8.95)	775	9 (1.2)	4.45 (1.49-13.2)
* χ*_1_^2^ *for trend*			*p < 0.001*			*p = 0.005*			*p = 0.007*
Number of births	1,056			955			3,225		
0	228	16 (7.0)	1	136	1 (0.7)	1	942	2 (0.2)	1
≥1	828	169 (20.4)	1.62 (0.86-3.04)	819	30 (3.7)	3.06 (0.39-24.1)	2,283	16 (0.7)	0.48 (0.06-3.82)

^a^Adjusted for age (5 years groups), as appropriate

**Fig. 1:**
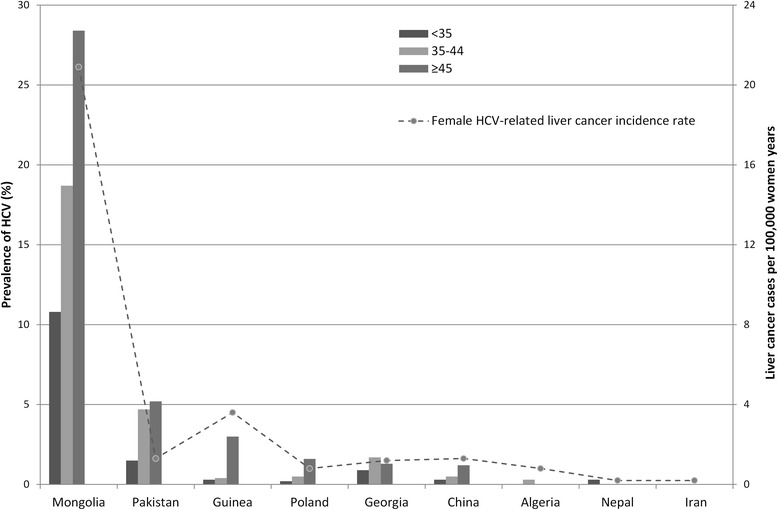
Correlation of age-specific HCV prevalence with HCV-related liver cancer incidence in women ≥45 years

Age-specific HCV prevalence estimates are shown by study country in Fig. 1, in order of highest to lowest HCV prevalence among women aged ≥45 years, and are plotted against country-specific estimates of female HCV-related liver cancer incidence rates. Age-specific increases in HCV prevalence were observable in Mongolia, Pakistan, Guinea, Poland and China. HCV prevalence estimates in women aged ≥45 years correlated well with female HCV-related liver cancer incidence rates, ranging between the extremes of Mongolia (28.4% HCV prevalence versus 20.9 cases of HCV liver cancer per 100,000 women) and Iran (0.0% HCV prevalence versus 0.2 cases of HCV liver cancer per 100,000 women). The only slight exception to this correlation was Pakistan, which showed a high HCV prevalence (5.2%) in comparison to a relatively low estimate of female HCV-related liver cancer (1.3 cases per 100,000 women).

## Discussion

Benefiting from a standardized population-based sampling protocol and a validated serological assay, we describe important variation in HCV seroprevalence in women around the world and robustly confirm the absence of a sexual transmission route for HCV infection. Crude population-based HCV prevalence ranged from 17% in Mongolia down to 0% in Iran and correlated with country-specific estimates of female HCV-related liver cancer incidence, which are also highest in Mongolia (20.9 per 100,000 women) and lowest in Iran (0.2 per 100,000 women) in women aged ≥45 years. Furthermore, when available, our estimates were compatible with previous population-based estimates of HCV prevalence in the evaluated countries.

The extreme HCV prevalence in Mongolia was expected and has been previously described [7]. Indeed, Mongolia has the highest burden of HCV-related liver cancer in the world [23], driven by a long-lasting epidemic due to iatrogenic transmission of HCV through mass vaccination campaigns (e.g. smallpox, polio [25], blood transfusions [26, 27]) and extensive use of injected treatments [28]. At the lower extreme, we confirm an anti-HCV prevalence of less than 1% reported from a wide-range of other population-based studies in Iran [29], where a low fraction of liver cancers are estimated to be attributable to HCV [30].

The relatively high HCV prevalence found in Karachi, Pakistan (~5% in women aged 45+ years) is similar to that in previous large population-based surveys in Karachi [31, 32] and nationwide in Pakistan [33]. Indeed, HCV was reported to be the predominant cause of liver cancer in Pakistan [34, 35]. Given this high HCV prevalence, however, current estimates of HCV-related liver cancer incidence in Pakistani women are lower than expected. This may be due to limitations in liver cancer incidence data for Pakistan [24], or to a more recent iatrogenic spread of the virus compared to, for instance, Mongolia. Indeed the proportion of HCV-positive hepatocellular carcinoma is still increasing in Pakistan [34].

HCV prevalence estimates were highly consistent across the three Chinese sites (0.4-0.6%) and compatible with estimates of 0.4-1.0% in large population-based surveys of women performed since 2006 [36–39]. HCV prevalence is especially low below age 45. Thus, improvements in transfusion practices implemented in China in the mid-1990s, notably prohibition of paid donations of plasma and blood that were associated with mass transmission of HCV and HIV, appear to have largely controlled the HCV epidemic. Indeed, population-based HCV prevalence [36], and proportion of liver cancer attributed to HCV [34], appears to be relatively low and decreasing over time in China.

HCV prevalence in women in Tbilisi, Georgia (1.3%) is compatible with that from a previous population-based survey in which the vast majority of HCV infections in Tbilisi were observed to occur among male intravenous drug users [40]. Indeed, increasing intravenous drug use is feared to be an important source of recent HCV transmission in many former Soviet republics [41]. Estimates of 0.8% HCV prevalence in Warsaw, Poland are similar to that in a nationwide study of 42,274 women (0.8% [42]), and with that modelled from meta-analytical data [43].

No population-based data are available for Algeria, but our HCV prevalence estimates (0.1%) compare well to the 0.2% and 0.6% reported among 1,000 [44] and 3,044 [45] pregnant women, respectively. This would suggest Algeria to have an HCV prevalence similar to its neighbors Morocco and Tunisia [46], and much lower than in Egypt [2], for which much more population-based data is available.

No population-based data are available for Nepal, but our HCV prevalence estimate of 0.2% compares well to the 0.1% reported among 2,007 female blood donors [47], suggesting that Nepal has largely escaped an epidemic of HCV to date.

Lastly, this study provides the first report on HCV infection in Guinea, for which the 0.9% HCV prevalence is lower than previous regional estimates for West sub-Saharan Africa (2.8% [1] and 5.3% [2]), which are nonetheless very sensitive to the lack of relevant data from this region of the world. Lower HCV prevalence among women aged under 45 years in Guinea may represent a cohort effect of reduced HCV transmission in younger generations.

HCV infection increased with age in most countries, presumably due to accumulating risk of exposure. This confirms steady increases up to the age of 60 years reported in a meta-analysis of age-specific HCV infection from Mongolia and Poland [43], and in large studies in China [36–39].

Given the orientation of the surveys towards studying HPV, questionnaires did not include variables that would be useful to studying specific iatrogenic transmission routes. Nevertheless, to our knowledge, this is the first study to suggest the existence of a possible association between HCV infection and parity, which persisted even after adjustment for center and age, which may represent a risk of HCV transmission by medical interventions/hospitalizations linked to childbirth. On the other hand, we were able to robustly confirm the absence of sexual transmission of HCV in the general female population, through the null association with number of sexual partners, and presence of cervical HPV infection, as proxies of sexual intercourse.

Our data arises entirely from females in selected towns, and may not be representative of males or other areas in the same country. In most populations around the world, HCV prevalence in females has been estimated to be similar or lower than that in males [43]. However, at least Mongolia [43] and China [38] may represent exceptions to this rule. In any case, we propose that our population-based sampling procedures provide a more representative picture than studies limited to typically young pregnant women or blood donors.

HCV genotypes are known to vary between the different worldwide regions represented by this study. Nevertheless, the cross-reactivity of serological assays for NS3 and Core proteins of genotypes 1a, 1b and 2a does not suggest that their performance should be affected by the existence of HCV serotypes [7]. Furthermore, the assay was shown to be highly reproducible when a large subset of samples were re-tested on a second occasion, and has been well-validated against commercial HCV tests [7] and for use with dried blood spots [19]. Taken together, we believe that this protocol represents a useful model for obtaining HCV prevalence data from low- and middle-income settings where data is scarce, for both men and women alike. Indeed, DBS do not require blood centrifugation and allow storage and shipment at ambient temperature, thus facilitating field work for seroepidemiological studies in environments with limited technical infrastructure, and the research serological assay is both high-throughput and cost-effective.

## Conclusions

HCV prevalence varied enormously across the female populations represented in our study. Medical interventions/hospitalizations linked to childbirth may have represented a route of HCV transmission, at least in some settings, but not sexual intercourse. Combining dried blood spot collection with high-throughput HCV assays can facilitate seroepidemiological studies in LMIC where data is otherwise scarce. Indeed, the availability of population-based HCV estimates is going to become increasingly relevant in the next years, as countries consider the cost and public health priority of going beyond the prevention of HCV transmission by ensuring safety of medical interventions, towards screen-and-treat approaches that can benefit from a new generation of highly performant oral HCV treatment regimens [5].

## Abbreviations

CI: confidence interval
DBS: dried blood spot
DKFZ: German Cancer Research Center
HCV: hepatitis C virus
HPV: human papillomavirus virus
IARC: International Agency for Research on Cancer
LMIC: low and middle-income countries
MFI: median R-phycoerythrin fluorescence intensity
PR: prevalence ratio
